# Highly Reproducible Surface-Enhanced Raman Scattering Detection of Alternariol Using Silver-Embedded Silica Nanoparticles

**DOI:** 10.3390/s20123523

**Published:** 2020-06-22

**Authors:** Eunil Hahm, Yoon-Hee Kim, Xuan-Hung Pham, Bong-Hyun Jun

**Affiliations:** Department of Bioscience and Biotechnology, Konkuk University, Seoul 05029, Korea; greenice@konkuk.ac.kr (E.H.); yoonhees@konkuk.ac.kr (Y.-H.K.); phamricky@gmail.com (X.-H.P.)

**Keywords:** reproducibility, SERS, alternariol

## Abstract

Alternariol (AOH) is a mycotoxin from fungi that has been found in processed foods due to its high thermal stability. To address the complexity and costs of conventional AOH detection methods, we propose an alternative based on surface-enhanced Raman scattering (SERS) and specially designed nanoparticle substrate. Herein, silver-embedded silica (SiO_2_@Ag) nanoparticles with a highly reproducible SERS signal were successfully developed for detecting AOH. Silica nanoparticles (~145 nm) were used as a template to deposit silver nanoparticles (~17 nm), thereby generating SiO_2_@Ag. The SiO_2_@Ag nanoparticles showed a good linearity between SERS signal intensity and AOH concentrations from 16 to 1000 nM with a limit of detection of 4.83 nM. Additionally, the SERS signal of the SiO_2_@Ag nanoparticles was highly reproducible, with relative standard deviations of 2.33–5.95% in the AOH concentration range from 10 to 10,000 nM, demonstrating the reliability of the proposed SERS method.

## 1. Introduction

Alternariol (AOH) is one of the major mycotoxins of the genus *Alternaria* in the kingdom fungi [1]. Several kinds of *Alternaria* species, which contaminate a wide variety of crops, have been reported [2]. Moreover, AOH has been detected in processed food products, such as bread, fruit juices, and wine, owing to its high thermal stability [3,4,5]. For the detection of AOH, various analytical methods have been proposed, including gas chromatography–mass spectrometry, liquid chromatography–mass spectrometry, and liquid chromatography–tandem mass spectrometry [6,7]. Although high-performance liquid chromatography is the most robust and reliable method, it requires a harsh solvent, high power source, complex pretreatment process, bulky and sophisticated operation, and trained personnel; furthermore, it is a time consuming and expensive method [8,9]. Therefore, a rapid, simple, highly sensitive, and stable alternative method should be developed for the detection of AOH.

Surface-enhanced Raman scattering (SERS) analysis, an analytical technique used to detect a variety of substances, is regarded as non-destructive, highly sensitive, and cost effective [10]. In general, the SERS phenomenon yields a powerful Raman scattering signal from a target molecule adsorbed on a nanostructured metal surface, wherein the signal is enhanced by ~10^6^ times compared with the typical Raman signal [11]. In particular, molecules located in nanoscale gap structures, which are known as hotspots, generate much more intense Raman signals since the local electromagnetic field is extremely enhanced [12]. Therefore, AOH detection using SERS was proposed to overcome the labor intensive and time-consuming weaknesses of the chromatographic methods. This is because SERS is highly sensitive and rapid and simple from the viewpoint of the analysis process [13]. According to the literature, pyridine-modified silver nanoparticles (Ag NPs) have been used as an SERS substrate for the detection of AOH. Although the limit of detection (LOD) of AOH was as low as 1.30 µg/L, the method based on Ag NPs alone requires improvement in quantitative detection due to aggregation and its lack of fabrication reproducibility [14,15].

Metal-NP-assembled silica NPs in a core-satellites structure have been reported and proven as reliable and robust SERS active substrates [16,17]. Metal-NP-assembled silica NPs exhibited a uniform shape and reproducible fabrication. The structure of the assembled metal NPs on a silica surface creates numerous hot spots due to nano-scale gap, indicating a high sensitivity of the metal-NP-assembled silica NPs.

In this study, a highly reproducible SERS signal was measured utilizing Ag-embedded silica NPs (SiO_2_@Ag NPs), one of metal-NP-assembled silica NPs, for determination of AOH. The SiO_2_@Ag nanostructure showed high sensitivity and good linearity for AOH detection, and the signal reliability was verified based on the high reproducibility of the SERS signal.

## 2. Materials and Methods

### 2.1. Materials

Tetraethyl orthosilicate (TEOS), (3-mercaptopropyl)trimethoxysilane (MPTS), ethylene glycol (EG), silver nitrate (AgNO_3_), octylamine, sodium hydroxide (NaOH), methanol (≥99.9%), AOH (~96%, from *Alternaria* sp.), and polyvinylpyrrolidone (PVP, average molecular weight ≈ 40,000) were purchased from Sigma-Aldrich (St. Louis, MO, USA). Aqueous ammonium hydroxide (NH_4_OH, 25%–28%) and ethanol (>99.9%) were purchased from Daejung Chemicals and Metals (Siheung, Korea). Water was purified using an EXL^®^5 S Bio Pure and Ultrapure water system (Vivagen, Seongnam, Korea).

### 2.2. Synthesis of SiO_2_@Ag NPs

The SiO_2_@Ag NPs were synthesized as previously reported with slight modifications [16]. First, SiO_2_ NPs were synthesized using the Stöber method [18]. TEOS (1.6 mL) and NH_4_OH (3 mL) were combined in ethanol (40 mL). After stirring for 20 h at 25 °C, the mixture was centrifuged at 8500 rpm for 15 min and washed several times by ethanol to remove the excess reagents. The obtained SiO_2_ NPs were dispersed in ethanol and adjusted to a concentration of 50 mg/mL.

To prepare thiolated SiO_2_ NPs (SiO_2_-SH), MPTS (200 µL) and NH_4_OH (40 µL) were added to the suspension of SiO_2_ NPs (200 mg) and stirred vigorously for 12 h at 25 °C. The mixture was washed several times and dispersed in ethanol. Ag NPs were introduced on the surface of the SiO_2_-SH NPs according to a modified polyol process. PVP (5 mg), as a capping agent, and AgNO_3_ (26 mg), were completely dissolved in EG (25 mL) to make separate solutions. SiO_2_-SH NPs (30 mg), AgNO_3_ in EG, and octylamine (41.4 µL) were added sequentially to the PVP EG solution. After stirring for 30 min, the suspension was centrifuged and washed several times by ethanol to remove unattached Ag NPs from the resulting SiO_2_@Ag NPs. Then, the SiO_2_@Ag NPs were dispersed in ethanol and stored at 25 °C.

### 2.3. Immobilization of AOH on SiO_2_@Ag NPs

A stock solution of AOH (1000 μM) was prepared by completely dissolving AOH in methanol and storing at −20 °C. The stock solution was diluted with methanol to prepare various concentrations of AOH standard solutions. Then, the prepared AOH standard solutions (500 μL) and SiO_2_@Ag NPs (0.25 mg in 500 μL of ethanol) were mixed and stirred vigorously at 25 °C. After stirring for 12 h, the mixture was centrifuged and dispersed in ethanol.

### 2.4. Transmission Electron Microscopy

Transmission electron microscopy (TEM) analysis was performed using a JEM-2100F field emission electron microscope (JEOL, Tokyo, Japan) operated at an acceleration voltage of 200 kV. TEM samples were prepared by evaporating a droplet of the NP suspension onto 400-mesh copper grid coated with carbon film.

### 2.5. UV-Visible Spectroscopy

UV-Vis absorption spectra of the colloidal NPs were measured from 300 to 800 nm using an Optizen POP single-beam type UV-Vis spectrophotometer (K Lab, Daejeon, Korea). The measurements were performed with 0.5 mg/mL NP suspensions in disposable polystyrene cuvettes.

### 2.6. SERS Measurements

SERS spectra were acquired with a Thermo Scientific DXR Raman microscope using a 532-nm laser at a power of 8.0 mW. The microscope was focused on the middle of a soda-lime glass capillary filled with a 1 mg/mL NP suspension. Data were collected with an exposure time of 16 s and generated with baseline correction to prevent spectral overlap with fluorescence. All measurements were repeated thrice, and the signals were averaged. The error bars of SERS intensity plots represent a 95% confidence interval.

## 3. Results and Discussion

### 3.1. Characterization of SiO_2_@Ag NPs

**Figure 1:**
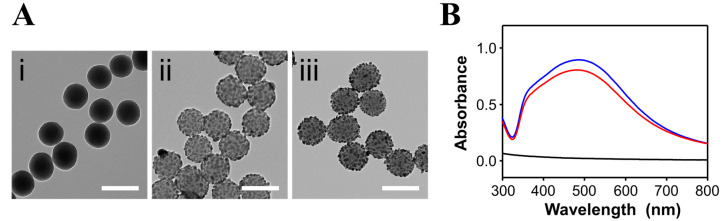
(**A**) TEM images and (**B**) UV-Vis absorption spectra of (i) SiO_2_ NPs, (ii) SiO_2_@Ag NPs, and (iii) SiO_2_@Ag NPs treated with a 10 µM AOH solution. Black, blue, and red lines in the absorption spectra correspond to (i), (ii), and (iii), respectively. The scale bars in (i–iii) are 200 nm.

SiO_2_@Ag NPs capable of generating enhanced SERS signals due to their numerous hotspots were synthesized for AOH detection. The silica core (~145 nm), which was synthesized using the Stöber method [18], was modified with thiol groups to enhance the metal affinity. Then, Ag NPs were adsorbed onto the surface of the silica cores by reduction. To identify the morphology of the NPs, TEM analysis was performed, and the sizes of the NPs were determined using Image J software. As shown in Figure 1A, the SiO_2_ and SiO_2_@Ag NPs were successfully synthesized with uniform sizes and shapes. The average diameter of the SiO_2_ NPs was 146 ± 5.3 nm. After the SiO_2_ NPs were thiolated to improve the metal affinity, Ag NPs with an average diameter of 17 nm were embedded densely onto the SiO_2_ NP cores. The embedded structure was maintained even after vigorous mixing with a methanolic solution of AOH.

The optical properties of the NPs were investigated by UV-Vis absorption spectroscopy (Figure 1B). The SiO_2_@Ag NPs showed a broad LSPR band from 325 to 800 nm with a maximum absorbance at 485 nm, whereas Ag NPs alone of a similar size show a maximum absorbance at ~400 nm [19]. This suggests that the Ag NPs were aggregated on the surfaces of the SiO_2_ NP cores [20].

### 3.2. SERS Activity of SiO_2_@Ag NPs for AOH Detection

**Figure 2:**
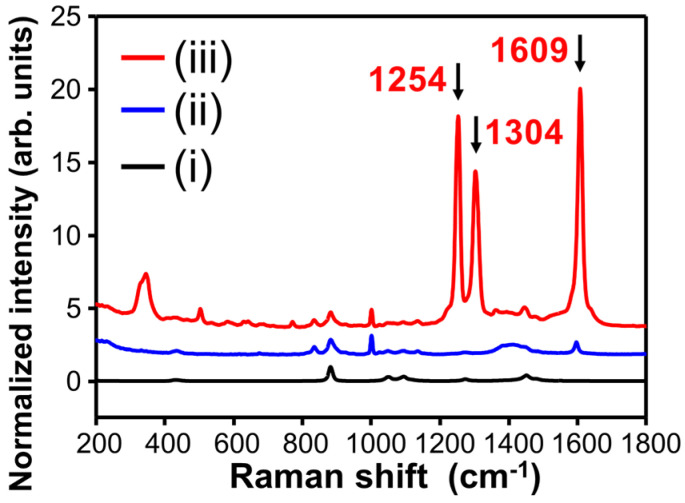
SERS spectra of (i) SiO_2_ NPs, (ii) SiO_2_@Ag NPs, and (iii) SiO_2_@Ag NPs treated with 10 µM AOH. All spectra are offset for the ease of comparison.

The Raman spectra of bare SiO_2_@Ag NPs and SiO_2_@Ag NPs reacted with AOH were measured to investigate the possibility of AOH detection. Figure 2 shows the SERS spectra of the AOH-treated SiO_2_@Ag NPs, which were normalized to the Raman intensity of the ethanol band at 883 cm^−1^. With the AOH treatment, several intense SERS bands appeared in the spectrum of the SiO_2_@Ag NPs. The most intense band at 1609 cm^−1^ was assigned to a C–C ring stretching mode [13,21,22], whereas the band at 1304 cm^−1^ was assigned to C–H ring stretching mode [13,22] and that at 1254 cm^−1^ was assigned to C–O–H and C–H in-plane bending [22,23]. These SERS bands are well matched with the reported Raman spectra of AOH [22]. Additionally, the characteristic SERS bands of AOH were maintained after washing the AOH-treated SiO_2_@Ag NPs with solvent (Figure S1). The Raman intensities of the characteristic bands of the AOH-treated SiO_2_@Ag NPs did not change significantly after washing with ethanol thrice, suggesting that the AOH molecules were stably adsorbed on the surfaces of the SERS-active SiO_2_@Ag NPs.

### 3.3. SERS Detection of AOH

**Figure 3:**
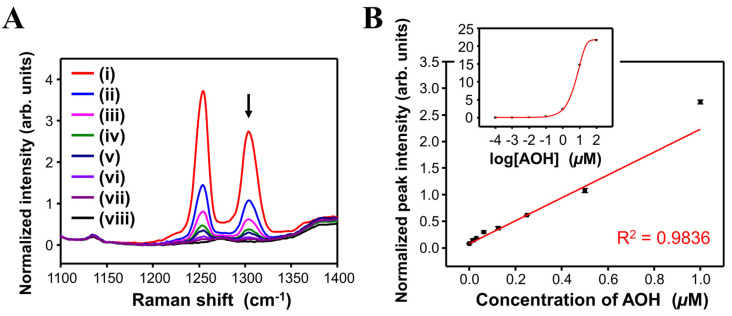
(**A**) SERS spectra and (**B**) SERS intensity plot at 1304 cm^−1^ of SiO_2_@Ag NPs treated with various concentration of AOH: (i) 1.000, (ii) 0.500, (iii) 0.250, (iv) 0.125, (v) 0.063, (vi) 0.031, (vii) 0.016, and (viii) 0 µM. Inset of (**B**): SERS intensity plot at 1304 cm^−1^ against logarithm of AOH concentration in the range of 10^−4^ to 10^2^ µM. The plot was fitted with an exponential curve to observe the linear working range.

Using the SiO_2_@Ag NPs as a SERS substrate, AOH detection was conducted, and the LOD was evaluated. Figure 3A shows the SERS spectra of SiO_2_@Ag NPs reacted with various AOH standard solutions from 16 to 1000 nM. The SiO_2_@Ag NPs reacted with AOH exhibited new peaks at 1254 and 1304 cm^−1^ compared with the control SiO_2_@Ag NPs. The intensity of the SERS bands at 1254 and 1304 cm^−1^ gradually decreased with AOH concentration, indicating that the SERS intensity of the AOH-treated SiO_2_@Ag NPs is proportional to the AOH concentration.

To evaluate the potential of the proposed detection method for quantitative analysis, the normalized SERS intensity at 1304 cm^−1^ was plotted according to the AOH concentration (Figure 3B). The relationship between SERS intensity and AOH concentration is suitably linear in the AOH concentration range from 16 to 1000 nM (correlation coefficient = 0.9836). The LOD was calculated as 4.83 nM at a signal-to-noise ratio of 3.3. Above an AOH concentration of ~100 µM, no linearity in the SERS intensity was observed (inset of Figure 3B). This may indicate that the SERS-active sites of the SiO_2_@Ag NPs were saturated. Similarly, the normalized SERS intensities at 1255 and 1609 cm^−1^ were also plotted according to AOH concentration, as shown in Figures S2 and S3, respectively. A good linearity between SERS intensity and AOH concentration was also observed for these SERS bands in the AOH concentration range from 16 to 1000 nM (correlation coefficients = 0.9837 and 0.9684 at 1255 and 1609 cm^−1^, respectively).

### 3.4. Reproducibility of SERS Intensity of SiO_2_@Ag NPs Reacted with AOH

**Figure 4:**
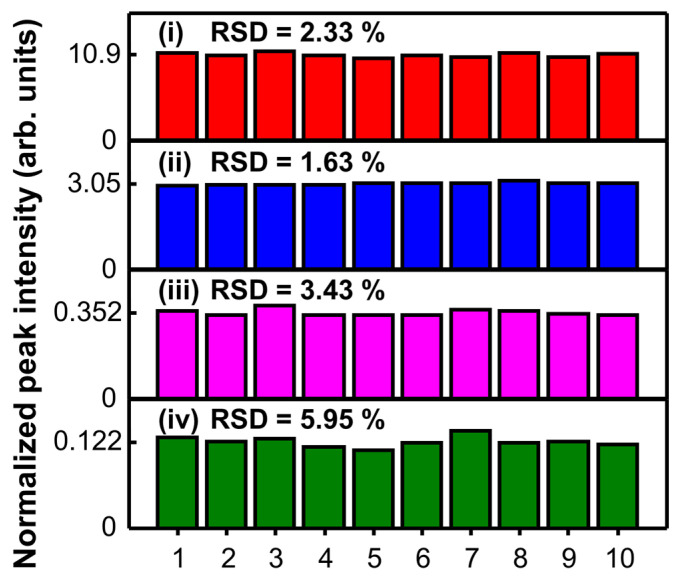
SERS intensities at 1304 cm^−1^ and corresponding relative standard deviations (RSDs) of SiO_2_@Ag NPs treated with (i) 10, (ii) 1.0, (iii) 0.10, and (iv) 0.010 μM AOH. Data were collected at different positions of a capillary loaded with the NP suspension. The average SERS intensity at each concentration is labeled on the *y*-axis.

To assess the SERS signal reproducibility of the proposed nanostructure, the SERS intensity of the SiO_2_@Ag NPs treated with various concentrations of AOH was recorded at 10 different sample locations. The SERS intensities at 1304 cm^−1^ of the SiO_2_@Ag NPs treated with AOH concentrations from 10 to 10,000 nM are shown in Figure 4. The calculated relative standard deviations (RSDs) indicated the reliability of the proposed SERS measurement through its reproducibility. Furthermore, the SERS intensity was measured for 10 batches of SiO_2_@Ag NPs reacted with 500 nM AOH. The results showed a low RSD (5.65%), as shown in Figure S4. These findings are attributable to the uniform SERS-active sites of the Ag NP-assembled SiO_2_ nanostructure.

## 4. Conclusions

In summary, SiO_2_@Ag NPs were prepared for the highly reproducible SERS detection of AOH. The sizes of the SiO_2_ NPs and Ag NPs present on the surface of the SiO_2_ NPs were 146 ± 5.3 nm and ~17 nm, respectively. As an application demonstration, the SiO_2_@Ag NPs were used as a substrate to detect AOH using SERS. The results showed a good linearity between the SERS signal and AOH concentration in the range from 16 to 1000 nM with an LOD of 4.83 nM. Additionally, the reproducibility of the SERS method using the SiO_2_@Ag NPs for AOH detection was demonstrated by low RSD values (2.33–5.95%) in the AOH concentration range from 10 to 10,000 nM, indicating the reliability of our technique. Therefore, the SiO_2_@Ag NPs which were verified the signal reliability of AOH are expected to be one of the promising sensor materials.

## Supplementary Materials

The following are available online at https://www.mdpi.com/1424-8220/20/12/3523/s1. Figure S1: SERS intensity at 1304 cm^−1^ of SiO_2_@Ag NPs added to 10^−6^ M AOH after washing with ethanol. Figure S2: SERS intensity plot at 1254 cm^−1^ of SiO_2_@Ag NPs treated with various concentration of AOH: (i) 1.000, (ii) 0.500, (iii) 0.250, (iv) 0.125, (v) 0.063, (vi) 0.031, (vii) 0.016, and (viii) 0 µM. Figure S3: SERS intensity plot at 1609 cm^−1^ of SiO_2_@Ag NPs treated with various concentration of AOH: (i) 1.000, (ii) 0.500, (iii) 0.250, (iv) 0.125, (v) 0.063, (vi) 0.031, (vii) 0.016, and (viii) 0 µM. Figure S4: Reproducibility of SERS intensity at 1304 cm^−1^ collected from 10 samples of different NP batches. The samples were prepared by treating each batch of SiO_2_@Ag NPs with 0.5 µM AOH. The relative standard deviation (RSD) was calculated as 5.65%.
