# Supplementary material for: Highly Reproducible Surface-Enhanced Raman Scattering Detection of Alternariol Using Silver-Embedded Silica Nanoparticles
Source: Sensors (Basel). 2020 Jun 22;20(12):3523. doi: 10.3390/s20123523 (PMC7349361; doi:10.3390/s20123523)
Supplement: Supplementary file 1 [file sensors-20-03523-s001.pdf]

sensors

Letter

Highly Reproducible Surface-Enhanced Raman Scattering Detection of Alternariol Using Silver-Embedded Silica Nanoparticles

Eunil Hahm †, Yoon-Hee Kim †, Xuan-Hung Pham and Bong-Hyun Jun *

Department of Bioscience and Biotechnology, Konkuk University, Seoul 05029, Korea; greenice@konkuk.ac.kr (E.H.); yoonhees@konkuk.ac.kr (Y.-H.K.); phamricky@gmail.com (X.-H.P.)

* Correspondence: bjun@konkuk.ac.kr; Tel.: +82-2-450-0521

† E.H. and Y.-H.K. contributed equally to this work.

Received: 28 May 2020; Accepted: 19 June 2020; Published: 22 June 2020

1. Supplementary.

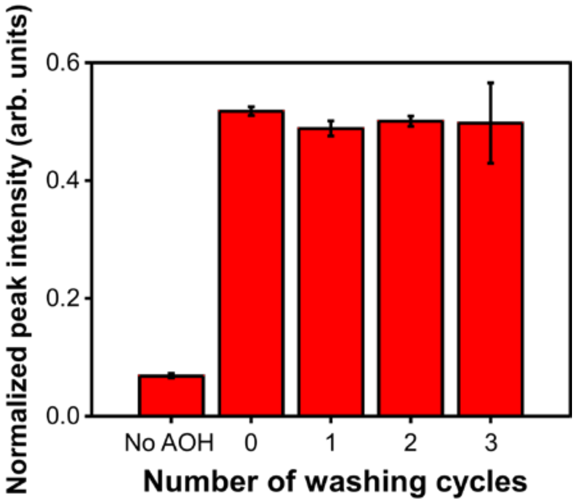

Figure S1. SERS intensity of SiO_2@Ag NPs added to 10^{-6} M AOH at 1304 cm^{-1} after washing with ethanol.

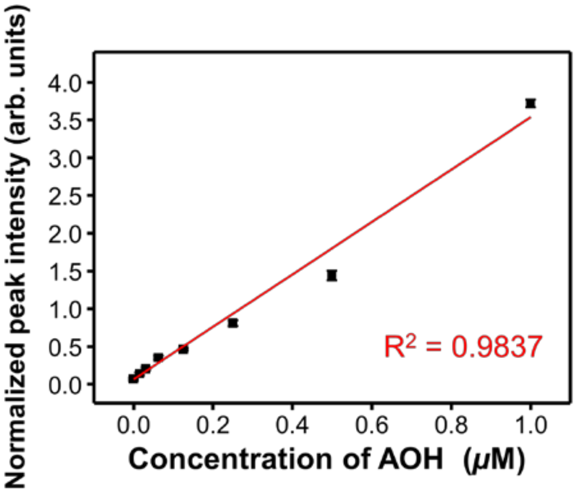

Figure S2. SERS intensity plot at 1254 cm^{-1} of SiO_2@Ag NPs treated with various concentrations of AOH: (i) 1.000, (ii) 0.500, (iii) 0.250, (iv) 0.125, (v) 0.063, (vi) 0.031, (vii) 0.016, and (viii) 0 μM.

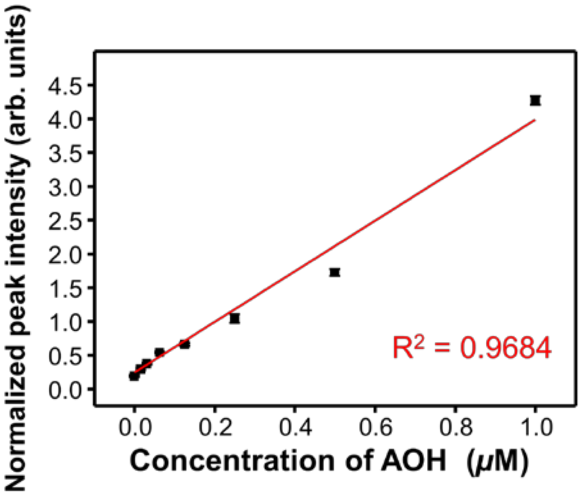

Figure S3. SERS intensity plot at 1609 cm^{-1} of SiO_2@Ag NPs treated with various concentrations of AOH: (i) 1.000, (ii) 0.500, (iii) 0.250, (iv) 0.125, (v) 0.063, (vi) 0.031, (vii) 0.016, and (viii) 0 μM.

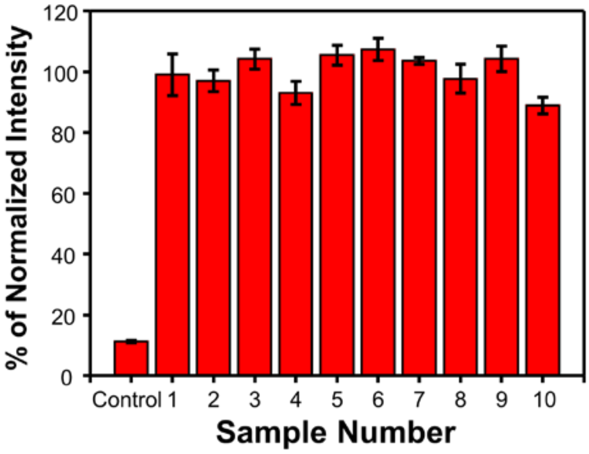

Figure S4. Reproducibility of SERS intensity at 1304 cm^{-1} collected from 10 samples of different NP batches. The samples were prepared by treating each batch of SiO_2@Ag NPs with 0.5 μM AOH. The relative standard deviation (RSD) was calculated as 5.65%.
